# Serial Thoracic Transforaminal Epidural Steroid Injections for Post-herpetic Neuralgia: A Case Report

**DOI:** 10.7759/cureus.21808

**Published:** 2022-02-01

**Authors:** Bao N Dinh, HuyKien Le, John Dinh, Joseph Mouhanna, Marcos A Sanchez-Gonzalez

**Affiliations:** 1 Family Medicine, Larkin Community Hospital, Miami, USA; 2 Pain Management, Larkin Community Hospital, Miami, USA; 3 Cardiology, Larkin Community Hospital, Miami, USA

**Keywords:** transforaminal steroid injection, neuropathic pain syndrome, shingles, varicella zoster virus, post-herpetic neuralgia

## Abstract

The chronic neuropathic pain of post-herpetic neuralgia (PHN) often persists for months or years after the acute herpes zoster (shingles) episode, may be severe and intractable, and can severely impact the overall quality of life. Antivirals, analgesics, and nerve blocks can effectively shorten the course of shingles and may help to prevent PHN. Although vaccination effectively prevents shingles and PHN, current therapies may be ineffective, and pain management can be challenging when PHN occurs. A 78-year-old female with severe PHN pain in the right thoracolumbar spine, right flank, and right lower abdomen showed poor responses to treatment with amitriptyline, gabapentin, and oxycodone/acetaminophen. However, a series of three thoracic transforaminal epidural steroid injections (TFESIs) effectively treated the PHN and achieved near-complete pain resolution. TFESI can be considered an early and first-choice treatment for PHN, but several courses may be required to achieve adequate and prolonged symptom control.

## Introduction

Post-herpetic neuralgia (PHN) is a chronic neuropathic pain syndrome characterized by chronic, persistent, and often severe pain following varicella-zoster virus (VZV) reactivation (shingles or herpes zoster) in the sensory ganglia of the cranial nerves or the dorsal root ganglion of the spinal cord. According to the CDC, approximately 99% of Americans born before 1980 have had chickenpox, and one in every three people will develop shingles in their lifetime. Of those who have had shingles, about one in 10 will develop PHN, the most common complication [1]. Herpes zoster is more common in the elderly and immunocompromised, and similarly, the risk of PHN increases with age. PHN is very rare in children, and the risk of PHN in adults younger than 60 is less than 2% [2]. However, PHN affects 15% of all patients who develop shingles and about half of the patients aged 60 years and over who develop shingles [3].

However, the pain of PHN can be challenging to manage, and oral therapies sometimes fail to control the pain adequately. Here we describe a case of persistent, intractable PHN pain successfully treated with a combination of oral analgesics and a series of transforaminal epidural steroid injections (TFESIs). This case allows us to describe the beneficial effect of TFESIs for resolving PHN and discuss mechanisms of pain in PHN and current management strategies.

## Case presentation

A 78-year-old woman with a past medical history of hypertension and arthritis presented with severe burning pain in the right thoracolumbar spine, right flank, and right lower abdomen in September 2020. She had presented to the ER in August 2020 with a rash and severe pain of shingles, at which time she was treated with oral antivirals, but her severe pain continued. She was started on gabapentin 300 mg and oxycodone/acetaminophen 5/325 mg three times a day since September 2020, but this failed to control the pain. An MRI of the thoracic spine showed normal alignment of the thoracic vertebrae, no compression fractures, normal discs, and patent spinal canal and foramina patent, thereby ruling out other thoracic spine causes for the pain.

Given the persisting pain, the decision was made to proceed with right thoracic TFESI at T10-11, T11-12, and T12-L1 under fluoroscopic guidance in November 2020. A solution of methylprednisolone 80 mg and 0.125% bupivacaine was equally divided and injected into each neuroforamen. One week later, the patient initially reported significant improvement in her pain but a gradual return to baseline after a few days. However, she was able to reduce her analgesics to oxycodone/acetaminophen 5/325 mg twice daily and gabapentin 300 mg three times a day.

A second right thoracic TFESI under fluoroscopic guidance was performed two weeks after the first block in the same manner and at the same spinal levels. One week later, the patient reported sustained relief of her abdominal and thoracic spine pain and a milder discomfort of muscular spasms and tightness, especially in the abdomen. She was, therefore, started on the non-steroidal anti-inflammatory drug meloxicam 15 mg daily and the muscle relaxant tizanidine 4 mg twice daily. Oxycodone/acetaminophen was discontinued, and tramadol 50 mg three times daily as needed was added for 15 days. Approximately two to three weeks after the second procedure, she reported muscle spasms but an overall improvement in pain and no need for taking opiates. Tizanidine was discontinued and was replaced with baclofen 10 mg twice daily due to the sedative effect. Gabapentin was increased to 600 mg three times daily with amitriptyline 25 mg before bed.

A third right thoracic TFESI under fluoroscopic guidance was performed about three months after the second block in the same manner and at the same spinal levels. This third procedure resulted in complete resolution of the skin sensitivity, and as a result, she was able to wear her normal (tight) clothes. A GI cause for abdominal spasm was excluded. Gabapentin was further increased to 800 mg three times a day, and physical therapy was started and continued for six weeks. Eventually, gabapentin and amitriptyline were gradually tapered and discontinued. Eight months after her symptoms onset in August 2020, her only regular medications were meloxicam 15 mg daily and a lidocaine patch, and she reported very minimal residual pain.

## Discussion

VZV infection, or chickenpox, is a common, highly contagious, benign, and self-limiting illness in immunocompetent hosts. It was nearly ubiquitous in children before the introduction of vaccination in 1995. It is followed by fever, malaise, pharyngitis, loss of appetite, culminating with a generalized vesicular rash, usually within 24 hours. It usually gets resolved within one to two weeks. The infection is predominantly controlled and maintained in an inactive state by cell-mediated immunity [4]. However, the decline in cell-mediated immunity seen in older individuals, immunosuppressed patients, and those with lymphoproliferative malignancies increases the risk of viral reactivation and shingles. Further proof is demonstrated in in vitro data where a reduction in VZV-specific T-cell frequency in the aging patient was associated with an increase in susceptibility to viral activation [5-7].

**Figure 1:**
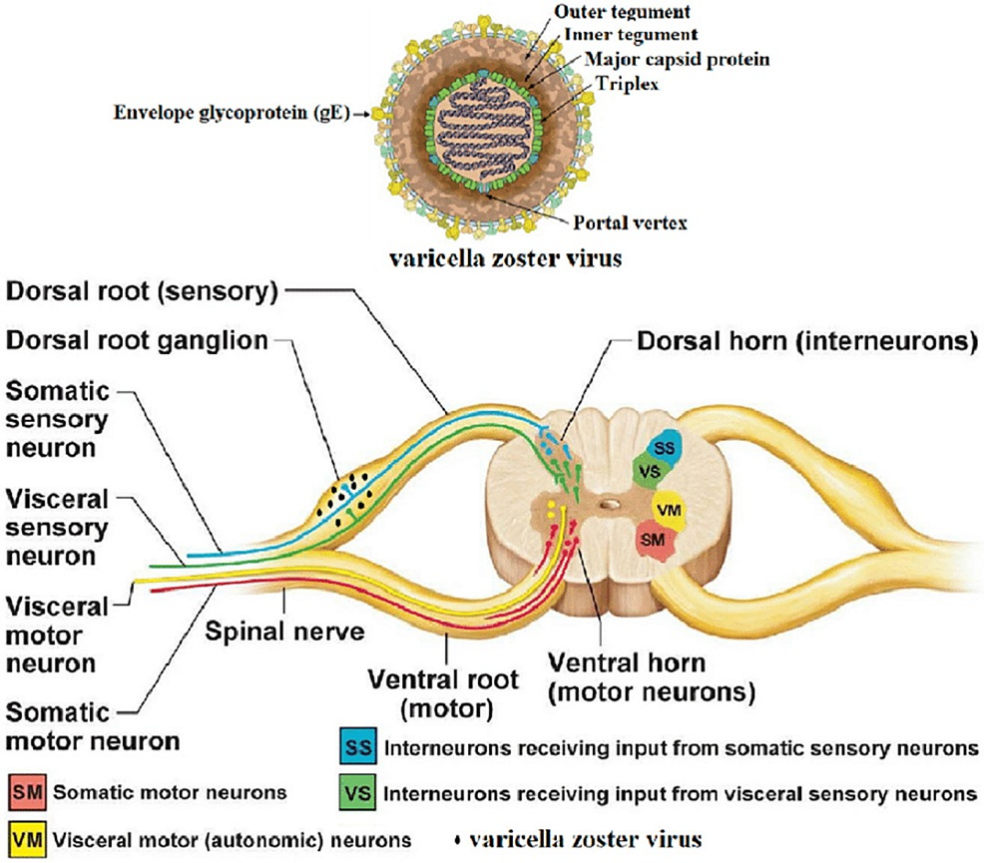
Varicella-zoster virus in the dorsal root ganglion. Source: [13]

PHN is a chronic sequela of shingles, and the pain can be intermittent or constant and is often described as burning, sharp, or stabbing [8, 9]. Over 90% of PHN patients develop allodynia, pain evoked by normally non-painful sensory stimuli [10]. Although the exact pathophysiology of PHN is uncertain, damage to the sensory nerves of the dorsal root ganglia and dorsal horns has been reported [11]. Two pathogenic mechanisms for PHN have been proposed: (1) nerve damage increases in the excitability of primary afferent neurons, thereby irritating nociceptors, resulting in central sensitization to cause pain and allodynia; and (2) deafferentation with central hyperactivity due to degeneration of nociceptive neurons resulting in pain without allodynia (Figure 1) [12-13].

Current pharmacological options for the management of PHN include topical analgesics (lidocaine and capsaicin), oral medications (gabapentinoids, tricyclic antidepressants [TCAs]), anticonvulsants, serotonin and norepinephrine reuptake inhibitors (SNRIs), opioid analgesics, and N-methyl-D-aspartate receptor antagonists [14-17]. In randomized control trials, gabapentin use was associated with pain reduction in up to 40% of patients [18], and pregabalin was associated with pain relief in up to 50% of patients [19]. In terms of TCAs, 47% of patients on amitriptyline reported moderate or greater relief [20], and up to 67% of patients treated with antidepressants experienced pain relief [21]. Topical analgesics such as lidocaine patches have been shown to relieve pain in up to 31% of patients [22], while 38% of patients experience pain relief with opioids [3].

Other modalities that have been used to treat PHN include transcutaneous electrical nerve stimulation (TENS), behavior therapy, blocks (epidural, intercostal nerve, and stellate ganglion), and steroid injections [15, 23]. In TFESI, steroids injected into the epidural space are hypothesized to relieve neuropathic pain by reducing deafferentation and inhibiting inflammation and swelling-induced neural ischemia. At the same time, local anesthetic provides analgesia to reduce the pain in the affected dermatomes [24]. TFESI has been reported to control PHN pain after only one injection [25, 26]. One small, short-term study reported no difference in analgesia between the interlaminar and transforaminal approaches for acute-phase shingles [27]. In addition, TFESI allows the medication to be delivered directly to the affected nerve root, thereby theoretically maximizing the therapeutic effects and limiting side effects. Continuous epidural analgesia has also been shown to be an effective analgesic in acute herpes zoster and PHN, increasing remission rates. In this approach, patients receive an initial bolus of steroids and local anesthetic at the affected spinal nerve(s) followed by continuous epidural infusion of a local anesthetic mixed with fentanyl [24]. Although not well studied, botulinum toxin has also been described for pain relief in PHN [28]. Spinal cord stimulation and subcutaneous peripheral nerve stimulation are considered last-resort treatments [29].

Despite these varied treatment options, the management of PHN remains suboptimal. About 40-50% of patients have persistent pain despite using multiple therapies [23]. In addition, early antiviral therapy does not significantly reduce the incidence of PHN [30]. Therefore, the CDC recommends herpes zoster vaccination for individuals aged 50 years or older to reduce the risk of developing herpes zoster and PHN [31], with two doses of recombinant zoster vaccine over 90% effective in preventing shingles and PHN.

Prior to treatment with TFESI, our patient was in severe pain despite taking opiates and non-opiate analgesics. Other thoracic pathologies were ruled out. Combined gabapentinoids and opioids provided only minimal pain relief. While the initial thoracic TFESI reduced her opioid intake, three TFESI procedures were required to achieve adequate, long-term, near-complete pain resolution without the need for anticonvulsants, TCAs, opioid analgesics, or gabapentinoids. The patient developed a short-lived spasm and tightness in the abdomen with the second injection. Although the cause of her symptoms is not known, GI pathology has been ruled out. Steroid-induced myopathy is also unlikely. Limitation includes gabapentin dosage increase to 800 mg TID which could contribute to improving her symptoms. TFESIs may represent an effective curative treatment for PHN, although a series of TFESIs may be required in some patients to achieve adequate control.

## Conclusions

Physicians should be aware of how prevalent neuropathic pain secondary to PHN is and how debilitating it can be to patients. Physicians and especially primary care physicians (PCPs) should be aware of the importance of diagnosing and referring patients with PHN early on to an interventionalist for consideration of TFESIs since up to 50% of patients have persistent pain despite the use of multiple therapies. The utilization of TFESIs contributes to reducing pain by affecting nerves and reducing deafferentation. Our case depicts the successful use of TFESIs, which may need to be administered several times to alleviate pain and resolve PHN.
